# Determination of Cellular Lipids Bound to Human CD1d Molecules

**DOI:** 10.1371/journal.pone.0005325

**Published:** 2009-05-05

**Authors:** Daryl Cox, Lisa Fox, Runying Tian, Wilfried Bardet, Matthew Skaley, Danijela Mojsilovic, Jenny Gumperz, William Hildebrand

**Affiliations:** 1 Department of Microbiology and Immunology, University of Oklahoma Health Sciences Center, Oklahoma City, Oklahoma, United States of America; 2 Department of Medical Microbiology and Immunology, University of Wisconsin School of Medicine and Public Health, Madison, Wisconsin, United States of America; 3 Department of Chemistry, Southern Nazarene University, Bethany, Oklahoma, United States of America; Weatherall Institute of Molecular Medicine, United Kingdom

## Abstract

CD1 molecules are glycoproteins that present lipid antigens at the cell surface for immunological recognition by specialized populations of T lymphocytes. Prior experimental data suggest a wide variety of lipid species can bind to CD1 molecules, but little is known about the characteristics of cellular ligands that are selected for presentation. Here we have molecularly characterized lipids bound to the human CD1d isoform. Ligands were eluted from secreted CD1d molecules and separated by normal phase HPLC, then characterized by mass spectroscopy. A total of 177 lipid species were molecularly identified, comprising glycerophospholipids and sphingolipids. The glycerophospholipids included common diacylglycerol species, reduced forms known as plasmalogens, lyso-phospholipids (monoacyl species), and cardiolipins (tetraacyl species). The sphingolipids included sphingomyelins and glycosylated forms, such as the ganglioside GM3. These results demonstrate that human CD1d molecules bind a surprising diversity of lipid structures within the secretory pathway, including compounds that have been reported to play roles in cancer, autoimmune diseases, lipid signaling, and cell death.

## Introduction

CD1 molecules are a family of β_2_-microglobulin associated glycoproteins that present lipids and glycolipids at the cell surface for recognition by T lymphocytes [1]. There are five CD1 isoforms (CD1a, CD1b, CD1c, CD1d, and CD1e). Of these, the CD1d isoform has attracted particular attention because it is the restricting element for a subpopulation of T cells, called Natural Killer T (NKT) cells, that has potent immuno-modulatory properties [2]. In addition to recognizing certain microbial lipids, NKT cells have been found to respond specifically to CD1d-mediated presentation of endogenous cellular lipids [2]. However, the self lipids recognized by human NKT cells remain poorly characterized. Similarly, the molecular principles that govern the selection of lipids that are presented by CD1d molecules for immunological surveillance are not well understood.

Crystal structures of murine CD1d have been solved with a variety of bound ligands, including glycosylated sphingolipids (GSLs), phosphatidylcholine (PC), and a mannosylated form of phosphatidylinositol (PI) [3]–[8]. Human CD1d molecules have been crystallized bound to the glycosylated sphingolipid α-galactosylceramide (α-GalCer) [9]. These analyses have revealed that CD1d molecules have an overall structure resembling MHC class I, but that their ligand binding groove is deeper and more hydrophobic. Lipid ligands bind to CD1d with their hydrophobic carbon chains largely buried within the binding groove, and their polar head groups accessible at the molecular surface. The crystal structures indicate there is some hydrogen bonding between the CD1d molecule and features of the polar head groups of bound ligands, however, it is not clear how much selection is exerted by the CD1d molecule on the chemical nature of the ligand's head group. Nevertheless, it is clear from the crystallographic data that a wide variety of polar head group structures can be accommodated.

In contrast, hydrocarbon chains of bound lipids usually fit snugly within the hydrophobic binding site. The CD1d ligand binding site contains two major pockets, named A′ and C′ for human CD1d [9] or A′ and F′ for murine CD1d [3], with the A′ pocket able to accommodate somewhat longer hydrocarbon chains (approximately 26 carbons) than the C′/F′ pocket (approximately 18 carbons). The crystallographic data suggest that hydrocarbon chains containing multiple double bonds bind preferentially in the A′ pocket where they can adopt a curved conformation, whereas the C′/F′ pocket provides a more linear configuration. However, biochemical analyses indicate that CD1d molecules can bind a diverse array of ligands that include substantial variation in radyl group structures [10]–[13]. The stability of lipid binding to CD1d molecules has been related to the length of the hydrocarbon chains and the manner in which they fill the binding groove, with longer chains that fit well into the A′ and C′ pockets of human CD1d providing for increased half-lives of association [11]. Nevertheless, it is important to note that antigenic lipids in which one of the hydrocarbon chains is severely truncated can still bind to CD1d molecules in a sufficiently stable manner to stimulate NKT cell responses [13], [14].

Previous studies have identified glycosylated phosphatidylinositols (GPIs) and unmodified PI as major mammalian cellular ligands of murine CD1d molecules [15], [16]. Material eluted from murine CD1d molecules that were purified from transfected insect cells included mainly PC and phosphatidylethanolamine (PE) [7]. Cellular ligands bound to human CD1d molecules included PI molecules with a variety of acyl chain structures [17]. However, while individual murine and human NKT cells have been identified that specifically recognize phospholipids such as PI and PE as antigens presented by CD1d, NKT cells that recognize these abundant phospholipids appear to be rare [18]–[20]. In contrast, most murine NKT cells recognize a particular tri-glycosylated sphingolipid called isoglobotrihexosylceramide (iGb3), which may be a much less abundant component of the pool of lipids presented by CD1d molecules since it is produced in lysosomes by degradation of a mature form that contains four sugars [21]. It is not clear whether iGb3 is also a self antigen for human NKT cells since humans lack the enzyme required to synthesize this compound [22], however it seems likely that the self antigens that stimulate human NKT cells may also be minor components of the pool of cellular ligands presented by human CD1d molecules. Thus, a high-resolution analysis of the ligands bound to human CD1d is critical for identifying compounds of interest that may regulate human NKT cell responses. Additionally, such an analysis would provide information about the structural characteristics of lipid species that are naturally selected for binding by CD1d. This information will be key for designing new compounds that can be used therapeutically to modulate immune responses by stimulating NKT cells, since such pharmacological lipid antigens will need to be able to compete effectively with endogenous lipids for binding to CD1d molecules on antigen presenting cells.

## Results

**Figure 1:**
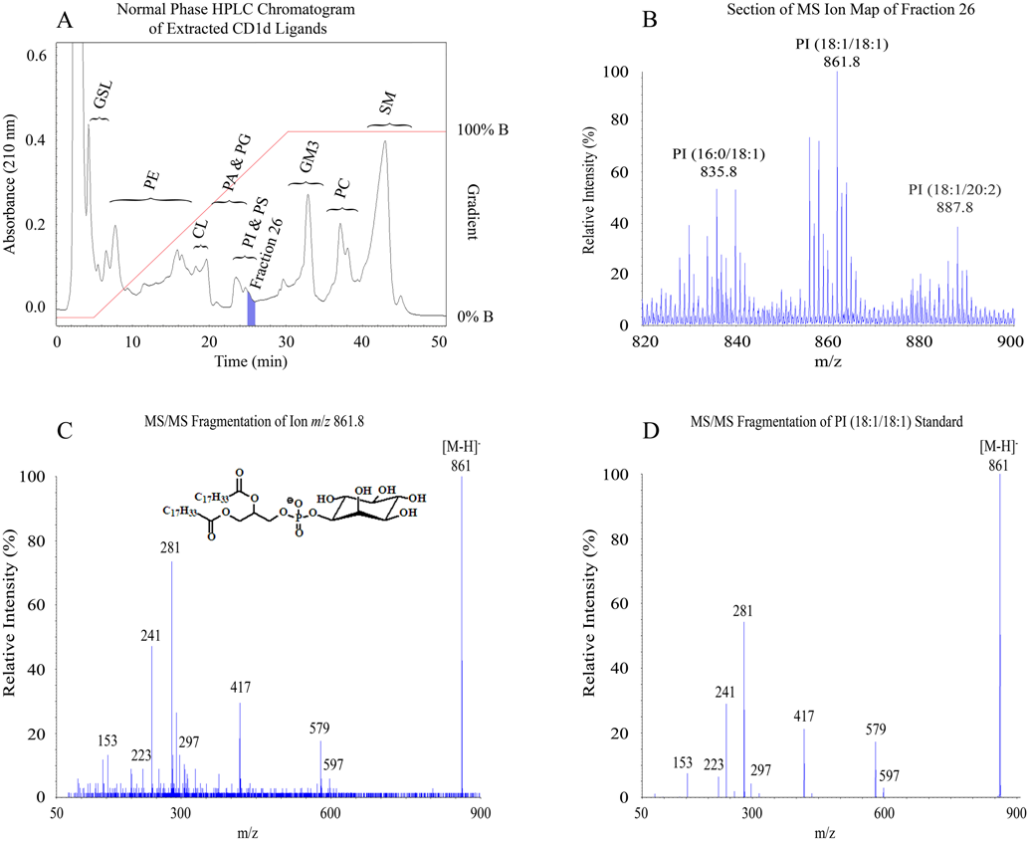
Isolation and characterization of ligands bound to human CD1d molecules. Secreted CD1d molecules were isolated by affinity chromatography and subject to organic extraction followed normal phase chromatographic separation (HPLC), and mass spectrometry (MS). A) HPLC chromatogram of extracted material. The general categories of lipids found along the profile are indicated using the following abbreviations: GSL, glycosphingolipid; PE, phosphatidylethanolamine; CL, cardiolipin; PA, phosphatidic acid; PG, phosphatidylglycerol; PI, phosphatidylinositol; PS, phosphatidylserine; GM3, ganglioside GM3; PC, phosphatidylcholine; SM, sphingomeylin. B) A section of MS ion map of fraction 26 in the negative ion mode. Ions were manually selected for MS/MS fragmentation. Within this section of the ion map three lipids were characterized, as shown by the labels above the corresponding peaks. C) MS/MS fragmentation pattern of one of these lipids, ion *m*/*z* 861. The [M−H]^−^ of ion 861 provides ions *m*/*z* 597 [M−H−C_16_H_31_CH = C = O]^−^ (the loss of the 18∶1 ketene), *m*/*z* 579 [M−H−C_17_H_33_CO_2_H]^−^ (the loss of the 18∶1 carboxylic acid), *m*/*z* 417 [M−H−C_16_H_31_CH = C = O−Ins]^−^ (the loss of the 18∶1 ketene and inositol), *m*/*z* 281 [C_17_H_33_CO_2_]^−^, (18∶1 fatty acid), *m*/*z* 241 [Ins-PO_3_−H_2_O]^−^ (dehydrated inositol phosphate). D) MS/MS fragmentation pattern of synthetic PI (18∶1/18∶1).

Secreted human CD1d molecules (sCD1d) were generated by truncating the transmembrane and cytoplasmic domains, and were expressed in the HLA class I deficient human lymphoblastoid cell line 721.221 [23], [24]. The cells were grown in hollow-fiber bioreactors and approximately 25 mg of sCD1d was purified by affinity chromatography. Bound ligands were released and separated from the sCD1d molecules by employing a chloroform:methanol:water extraction protocol similar to that previously described by Bligh and Dyer [25]. The eluted material was further purified by normal phase HPLC, resulting in the fractionation of various classes of lipids within the ligand pool (Figure 1A). Fractions from the HPLC purification were subject to mass spectrometry (MS) in both positive and negative ion modes, generating ion maps of the compounds within each fraction (Figure 1B). Individual ion peaks from these maps were further analyzed by MS/MS, producing fragmentation patterns that allowed structural identification of the major species in the peak by comparison to previously published data and analysis of synthetic lipid standards (Figures 1C and 1D).

### Types of lipids bound by CD1d

**Figure 2:**
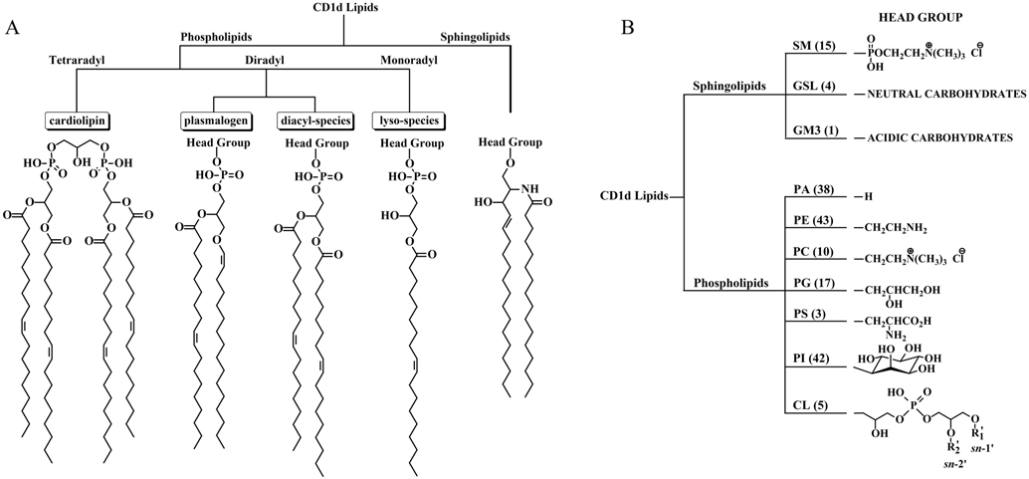
Overview of the diversity of lipids identified in the CD1d ligand pool . A total of 177 molecular species were identified. The majority of lipids characterized were phospholipids, but sphingolipid species were also identified. A) Tree diagram showing the categories and structural characteristics of lipids found within the ligand pool. B) Tree diagram showing the diversity of lipid head groups. Lipid classes are abbreviated as described in the legend for figure 1A. Numbers in parentheses represents the number of lipid species (i.e. radyl group carbon chain variations) characterized in that subcategory.

Lipids found within the sCD1d ligand pool varied in the backbone structure of the lipid and the nature of the lipid head group, and in the numbers, types, chain lengths and degree of unsaturation of the radyl groups. Two major categories of lipids were present: glycerophospholipids and sphingolipids (Figure 2). Within the glycerophospholipid category, three types of radyl group configurations were identified: diradyl species (i.e. containing two carbon chains) were predominant, but monoradyl lyso-phospholipids and tetraradyl cardiolipins were also found (Figure 2A). The diradyl species included common diacylglycerol phospholipids, as well as more unusual reduced forms called plasmalogens (Figure 2A). A variety of polar head groups were found among the glycerophospholipid species in the ligand pool (Figure 2B), including unmodified phosphatidic acid (PA), phosphatidylglycerol (PG), phosphatidylserine (PS), and species that have been previously identified in biochemical analyses as CD1d ligands such as PI, PE, and PC [7], [15]–[17]. Three classes of sphingolipid were also present in the ligand pool: sphingomyelin (SM), and glycosylated sphingolipids (GSL) containing either neutral or acidic modifications (Figure 2B). The sphingolipids included derivatives of sphingosine (i.e. ceramides) as well as forms derived from the structurally related base sphinganine. Notably, we were not able to determine the structures of a number of ions visible on the MS maps. These undetermined species may account for up to one-quarter of the ligand pool.

### Characteristics of lipid tails

**Figure 3:**
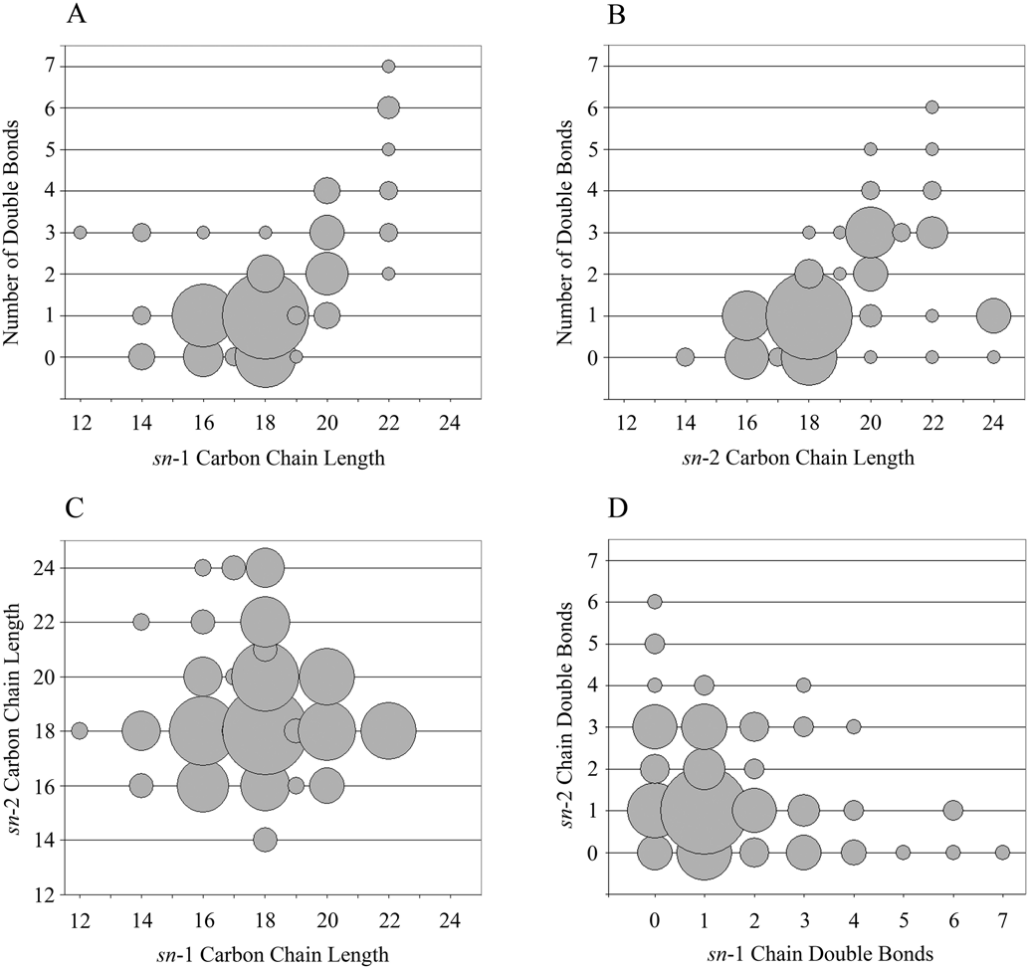
Structural characteristics of radyl group carbon chains. Analysis of the length and degree of unsaturation of the carbon chains of diradyl lipid species identified within the ligand pool. The sizes of the circles are proportional to the number of lipid species at each set of coordinates. A total of 149 lipid species were included in the analysis. A) Analysis of *sn*-1 carbon chains, comparing the number of double bonds to the length. B) Analysis of *sn*-2 carbon chains, comparing the number of double bonds to the length. C) Comparison of the lengths of the *sn*-2 versus *sn*-1 carbon chains. D) Comparison of the number of double bonds in the *sn*-2 versus *sn*-1 carbon chains.

The diversity of lipid head group structures found within the ligand pool is consistent with this feature exerting little influence on selection for binding to CD1d. In contrast, structural characteristics of the radyl groups of lipids are thought to play a major role in lipid binding to CD1 molecules. Previous crystallographic studies have suggested that in order to fit into the A′ and C′ pockets of CD1d molecules the carbon chains of diacylated lipids should not be longer than C26 and C18, respectively. To gain insight into the characteristics of lipids selected for binding to human CD1d, we analyzed the length and numbers of double bonds of carbon chains from diacylated ligand species. A total of 149 species were included in the analysis. *Sn*-1 chains ranged from 12–22 carbons and contained from 0–7 double bonds (Figure 3A). Most *sn*-1 chains (62%) were 16–18 carbons long and contained zero or one unsaturation (Figure 3A). *Sn*-2 chains ranged from 14–24 carbons and contained 0–6 double bonds (Figure 3B). Similar to the *sn*-1 chains, most *sn*-2 chains (58%) were 16–18 carbons with zero or one double bond (Figure 3B). In both cases, there was a tendency for chains longer than 18 carbons to contain at least one double bond, and particularly among the *sn*-1 chains there was a clear positive correlation between longer chain lengths and higher numbers of double bonds (Figures 3A and 3B).

Based on the sizes of the A′ and C′ pockets observed in the human CD1d crystal structure [9], it might be predicted that diacylated lipids having both radyl chains longer than 18 carbons would not fit well into the CD1d binding site. Our data is quite consistent with this prediction, since out of 179 species that were molecularly characterized, only four had two chains longer than 18 carbons (i.e. C20∶3/C20∶3 PA, C20∶4/C20∶3 PA, C20∶3/C20∶3 PI, and C20∶3/C20∶4 PI), and in these cases the chains were only 20 carbons long and all contained 3 or more double bonds. With the exception of these four ligands, *sn*-1 or *sn*-2 chains longer than C18 were always paired with chains that were 18 carbons or less (Figure 3C). A comparison of the number of double bonds in each chain revealed that fully saturated lipids were rare in the CD1d ligand pool (4%), and species that contained one double bond in each chain were the most frequent type, comprising 46% of the total (Figure 3D). S*n*-1 and *sn*-2 chains that contained two or more double bonds were usually paired with chains that contained zero or one double bond (Figure 3D).

### Unexpected species: plasmalogens, cardiolipins, and lyso-phospholipids

**Figure 4:**
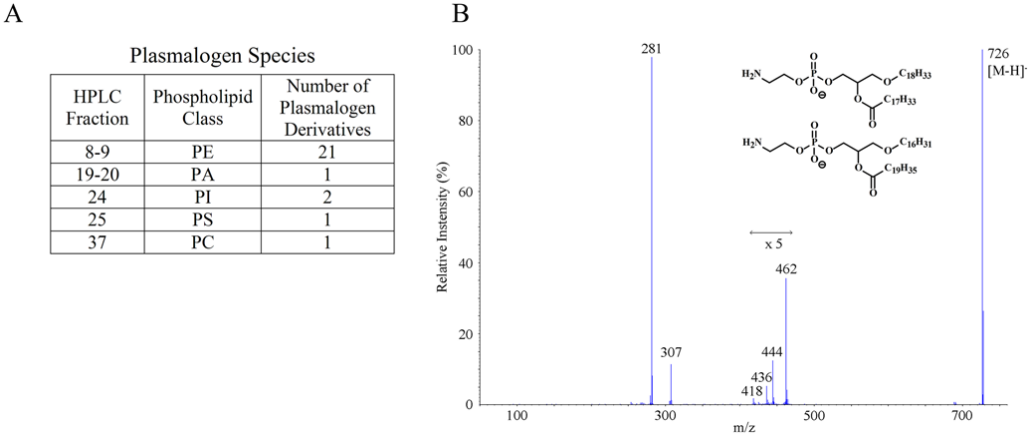
Identification of plasmalogens. A fraction of the diradyl phospholipid species within the CD1d ligand pool were identified as plasmalogens, as exemplified by the mass spectrometry data shown here. A) Table summarizing the types of plasmalogen phospholipids identified. B) Negative ion MS/MS of two PE ether plasmalogen species (*m*/*z* 726.7), from the MS ion map of Fraction 9. Only the carboxylate ion was characterized. Ions *m/z* 462 and *m/z* 444 result from the loss of the 18∶1 ketene and the 18∶1 carboxylic acid respectively; ions *m/z* 436 and *m/z* 418 result from the loss of the 20∶2 ketene and the 20∶2 carboxylic acid respectively. The molecular formulas for the resulting ether moieties was deduced from the mass of the [M−H−ketene]^−^ and [M−H−RCO_2_H]^−^ ions. The structures of the ether moieties were not unequivocally determined. They could be plasmenyl or plasmanyl radyl groups.

Previous biochemical analyses have suggested that common diacylglycerol phospholipids are major cellular ligands of CD1d [7], [15]–[17], but have provided little insight into less common ligands. Our high-resolution analysis permitted identification of a number of interesting but less abundant ligands of human CD1d. For example, we observed that a significant number of plasmalogen derivatives were included among the glycerophospholipids. These compounds contain either an ether (-CH_2_-CH_2_-O-) or vinyl ether (-CH = CH-O-) linked to the glycerol backbone at the *sn*-1 position instead of the usual ester (R^1^CO_2_-) linkage (Figure 2A), and are thought play a number of specialized biological roles [26]. Almost half of the PE species characterized fit into this category (21 out of the 43 total species of PE), while plasmalogen forms were uncommon among the other types of glycerophospholipids (1 PA, 1 PC, 1 PS, and 2 PI plasmalogens were found). Figure 4 shows MS/MS results identifying two PE plasmalogen ether species.

**Figure 5:**
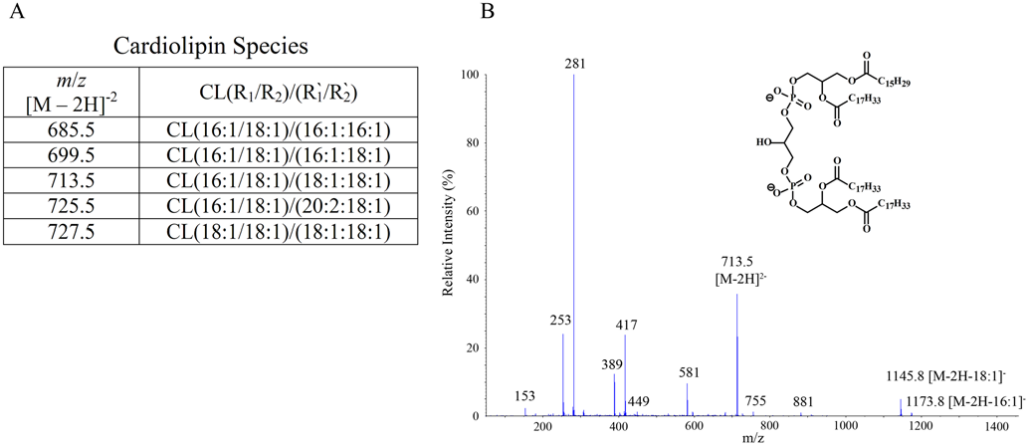
Identification of cardiolipins. Cardiolipins, which are tetraradyl dimers of phosphatidylglycerol, were present in the CD1d ligand pool. A) Table summarizing the cardiolipin species identified. B) Negative ion MS/MS of ion *m*/*z* 713.5 from the MS ion map of Fraction 18, containing cardiolipin (18∶1/16∶1)/(18∶1/18∶1). The map shows *m/z* 713.5 [M−2H]^2−^, *m/z* 1173.8 [M−2H−C_15_H_29_CO_2_]^−^, *m/z* 1145.8 [M−2H−C_17_H_33_CO_2_]^−^, *m/z* 581 [(713.5×2−C_16_H_31_CH = C = O)/2]^2−^, *m/z* 417 (loss of FA from *sn*-2 and *sn*-1 positions of 18∶1/18∶1 PA structure and the *sn*-1 position of 18∶1/16∶1 PA structure), *m/z* 389 (loss of FA from *sn*-2 position of 18∶1/16∶1 PA structure).

Cardiolipins are glycerophospholipids that are abundant in mitochondrial membranes, and are essentially composed of two phosphatidylglycerols linked together at the head group (Figure 2A). As the previously characterized CD1d ligands are mainly diacylated lipids, these tetraradyl compounds may seem unlikely candidates for cellular antigens. However, we found that cardiolipins were present in the CD1d ligand pool, and structurally identified five species (Figure 5A). Shown in Figure 5B is the MS/MS fragmentation pattern of a cardiolipin ion from fraction 18 of the HPLC separation of the CD1d ligand pool.

**Figure 6:**
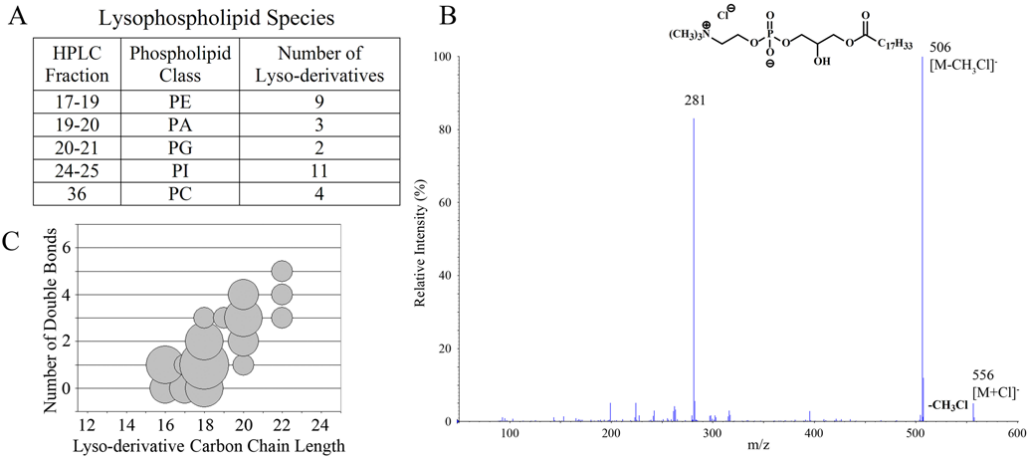
Identification of lysophospholipids. A variety of lyso-phospholipids (i.e. monoradyl species) were found in the CD1d ligand pool. A) Table summarizing the types of lyso-phospholipids identified. B) Negative ion MS/MS of ion *m*/*z* 556.5 from ion map of Fraction 36, containing the chloride ion adduct of C18∶1/C0∶0 PC (lyso-PC or LPC). Ion *m/z* 556 [M+Cl]^−^, gives rise to *m/z* 506 from the neutral loss of chloromethane ([M+Cl]^−^−CH_3_Cl). The fatty acid 18∶1 is indicated by the ion *m/z* 281. C) Analysis of the carbon chains of the identified lyso-phospholipid species for the number of double bonds compared to chain length.

Recent data has suggested that lipids containing only one hydrocarbon chain may serve as ligands for CD1d, and may thus stimulate T cell responses [14], [27], [28]. A lyso-derivative of the central nervous system lipid sulfatide was found to be recognized by a CD1d-restricted murine T cell line, and lyso-phosphatidylcholine (LPC) was reported to be recognized by CD1d-restricted T cells that are expanded in the blood of human multiple myeloma patients [27], [28]. In this analysis we found a considerable number of lyso-derivatives in the CD1d ligand pool (Figure 6A). Figure 6B shows the MS/MS pattern identifying a species of LPC. The radyl groups of the lyso-derivatives in the CD1d ligand pool showed a similar distribution of carbon chain lengths and double bonds to those of the *sn*-1 chains of the diacylated lipid species, although carbon chains shorter than C16 were not observed among the lyso- species (Figure 6C).

### Glycosylated lipids

**Figure 7:**
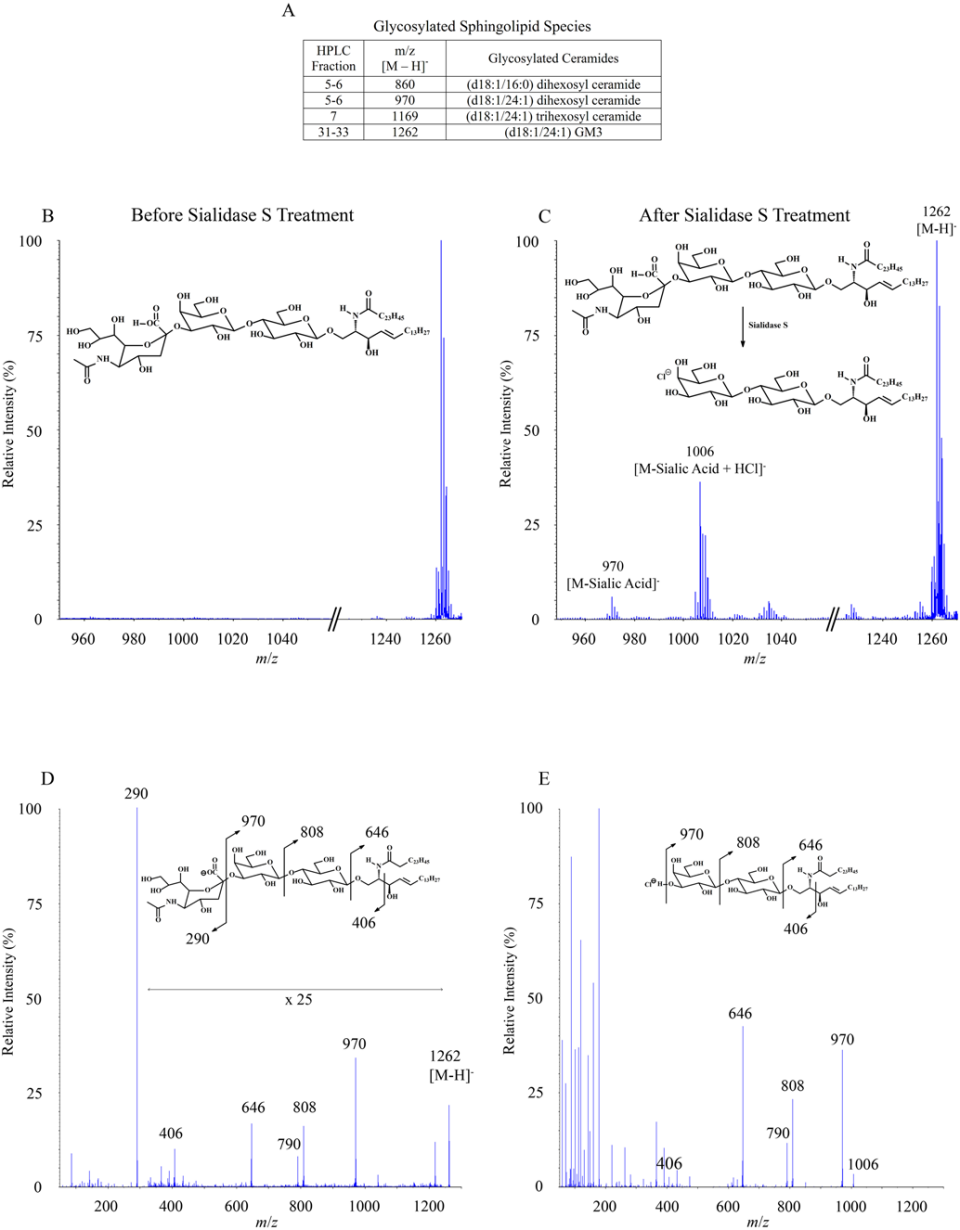
Identification of glycosylated sphingolipids. Four glycosylated sphingolipid species were identified in the CD1d ligand pool. The most abundant species was identified as the ganglioside GM3 (see Figure 1A). Additionally, three glycosylated ceramides were found. A) Table summarizing the glycosylated lipid ligands identified. B) The most abundant species, fraction 33 of the normal phase HPLC separation, was subject to further characterization by enzymatic digestion and mass spectrometry. Shown is the partial negative ion MS map of fraction 33. C) Partial negative ion MS map of fraction 33 after Sialidase S treatment. Note the formation of ions resulting from the cleavage of sialic acid from the parent compound. D) MS/MS fragmentation of ion *m*/*z* 1262 [M−H]^−^ from panel A. The fragmentation pattern shows the loss of sialic acid, *m*/*z* 970 [M−sialic acid]^−^, *m*/*z* 808 [M−sialic acid−hexose]^−^, *m*/*z* 790 [M−sialic acid−hexose−H_2_O]^−^, *m*/*z* 646 [M−sialic acid−hexose−hexose]^−^, *m*/*z* 406 [C_26_H_48_NO_2_]^−^ (N-acyl chain+carbons 1 and 2 of LCB). E) Negative ion MS/MS fragmentation of ion *m*/*z* 1006 [M+Cl]^−^ from panel C. The fragmentation pattern shows that after enzymatic cleavage of the sialic acid residue, the remaining two sugar residues are lost in an identical fashion to that of the GM3 species in Panel D.

Most of the compounds that have been found to be antigens for CD1d-restricted T cells are glycolipids [29]. Thus cellular glycolipids that are presented by CD1d molecules are of great interest. Several glycosylated lipids were identified within the ligand pool, all of which were GSLs containing oligosaccharides attached to a ceramide backbone (Figure 7A). The most abundant of these was identified as a derivative of the ganglioside GM3 by employing MS analysis before and after enzymatic digestion. The MS ion map of fraction 33 from the normal phase HPLC separation of the CD1d ligand pool revealed one major species (Figure 7B). Sialidase S treatment of fraction 33, which specifically releases terminal α(2-3)-linked sialic acids from a complex carbohydrate, resulted in the formation of ions produced by the cleavage of sialic acid from the parent compound (Figure 7C). The MS/MS fragmentation pattern of the ion from fraction 33 showed the distinctive initial loss of sialic acid followed by the stepwise loss of two hexoses, and also delineated ions corresponding to the ceramide backbone (Figure 7D). The MS/MS fragmentation pattern after sialidase S treatment showed the same stepwise loss of the remaining two hexoses (Figure 7E). The structural assignment of the ion from fraction 33 as a derivative of GM3 was confirmed by comparing it to the MS/MS fragmentation pattern of a purified GM3 standard (data not shown).

Additionally, three neutral GSLs were found in the CD1d ligand pool (Figure 7A). Two of these were assigned as di-hexosylceramides and the third as a tri-hexosylceramide based on their MS/MS fragmentation patterns, which clearly showed the sequential loss of all the hexoses to yield the ceramide (data not shown). We were not able to definitively determine the identities of the sugars found in these GSLs or the chemistry of their linkages. Also undetermined was whether glycosylphosphatidylinositols (GPIs) were present in the ligand pool. A previous analysis identified GPIs as a major constituent of the cellular ligands bound to murine CD1d [16], and thus this may also be a natural ligand of human CD1d molecules. It is worth noting that much of the material within the human CD1d ligand pool that we were not able to identify appeared to be of relatively high molecular weight, and therefore it is possible that more glycosylated ligands are present, and that these may include GPIs.

## Discussion

Like MHC class I molecules, CD1d molecules are synthesized in the endoplasmic reticulum (ER) and then follow the secretory route through the Golgi and out to the cell surface [30]. However, similar to MHC class II molecules, CD1d molecules are re-internalized from the cell surface and traffic through the endosomal vesicular system before exiting again to the cell surface [30]. Thus, CD1d molecules may acquire ligands in the secretory pathway, or within the endosomal system, or while they are on the cell surface. Since the secreted CD1d molecules that we have analyzed here traffic through the secretory pathway but not through the endosomal vesicular system, the pool of ligands bound to these CD1d molecules may not encompass all of those presented by wild type cell surface CD1d molecules. However, we have found that in contrast to the murine system, endosomal trafficking of human CD1d molecules is not required to stimulate autoreactive responses from human NKT cells [31]. Therefore, antigens that can activate human NKT cells are likely to be present among the cellular CD1d ligands identified in this analysis. Consistent with this, we show in a companion manuscript (see Fox et al., submitted concurrently) that many human NKT cells recognize LPC, a lipid that we show here was present in the CD1d ligand pool.

It is a concern that non-physiological lipids derived from the culture medium used to grow the human lymphoblastoid cells may load into the secreted CD1d molecules and thus become part of the ligand pool analyzed here. To address this point, we performed an extraction of a sample of the fetal bovine serum (FBS) that is diluted into a nutrient solution to make up the culture medium, and analyzed the lipids by HPLC and MS. The HPLC profile of the FBS lipids showed some peaks that overlapped with those from the CD1d ligand pool (see Figure S1), however MS analysis of fractions containing these peaks did not show any species in common (data not shown). This supports the conclusion that the ligands identified here were derived from the human lymphoblastoid cells used to produce the secreted CD1d molecules.

By characterizing a large number of molecular species within the CD1d ligand pool, our analysis provides new insights into the structural characteristics of ligands that are selected for binding to human CD1d. One area of interest concerns how the structural features of lipids influence their positioning within the binding site. Crystal structures of sphingolipids bound to murine or human CD1d molecules consistently show that the fatty acyl chain binds in the A′ pocket and the sphingosine chain is bound in the C′/F′ pocket [3]–[6], [9]. This lipid orientation appears not to be determined by the relative lengths of the two chains, since lipids containing very short fatty acyl chains are positioned in the same orientation as those in which it is longer. However, comparison of the two crystal structures of CD1d molecules containing bound phospholipids suggests that these ligands can bind in either of two orientations [7], [8]. In one case (murine CD1d with bound PC) the *sn*-1 chain, which is 12 carbons long, is bound in the F′ pocket and the *sn*-2 chain, which is 24 carbons long, is bound in the A′ pocket. In the other case (murine CD1d with mannosylated PI) the orientation of the lipid is flipped: the 16 carbon *sn*-1 chain binds in the A′ pocket, and the *sn*-2 chain, also 16 carbons long, binds in the F′ pocket. Assuming that the total hydrocarbon chain length that can fit into the C′/F′ pocket is about 18 carbons, our data is consistent with the possibility that phospholipids can bind in either orientation, since lengths greater than 18 carbons were observed for both the *sn*-1 and *sn*-2 chains, suggesting that either one can be placed in the larger A′ pocket. Moreover, longer *sn*-1 or *sn*-2 chains were almost always paired with chains of 18 carbons or less, which is consistent with a requirement for the other chain to fit in the C′ pocket. Considering that the orientation of the lipid within the CD1d binding site is likely to affect the positioning of the head group, these data suggest the surprising possibility that a single type of phospholipid might present two different epitopes for TCR recognition depending on the orientation of binding within the CD1d groove.

Another area of interest is the range and diversity of ligand species presented for immune surveillance by CD1d molecules. Our data indicate that human CD1d molecules bind several unexpected types of lipids within the secretory pathway, and these observations have a number of interesting implications. For example, we found that plasmalogen ethers, particularly of PE, accounted for a considerable fraction of the total diacylglycerol phospholipid species. Plasmalogens have been found to play an important role in maintaining proper cellular membrane functioning and in preventing oxidative damage [26], [32]. Because of their ether structure plasmalogens are more acid labile than other glycerophospholipids, and therefore they may be more likely to undergo cleavage within CD1d molecules if transported to acidic endosomal compartments. This might cause them to be more easily removed and replaced by other lipids, or might create new antigenic epitopes if lyso-phospholipid cleavage products remain bound to CD1d.

The finding that cardiolipins were present within the CD1d ligand pool was surprising for two reasons. First, it is not clear how such tetraradyl species would bind to a single CD1d molecule, as the crystallographic data suggest there is only room for two hydrocarbon chains within the binding groove. We speculate that cardiolipins may bind concurrently to two CD1d molecules and thus ligate them together, though further analysis will be required to determine this. The second surprising aspect of this observation is that cardiolipins are confined almost entirely to the inner membrane of the mitochondria where they are synthesized from diacylated precursor phospholipids [33], and therefore their presence in the CD1d ligand pool suggests that they are transported from mitochondria to a location that permits loading into CD1d molecules, such as the ER. Notably, the finding that CD1d molecules may access lipids from mitochondria is consistent with a previous survey of peptide ligands eluted from the MHC class I molecule HLA-B*1801, in which we found that a number of endogenously loaded peptides were derived from proteins localized to the mitochondria [34]. Thus, much like their classical HLA relatives, CD1d molecules survey ligands that originate in diverse intracellular compartments.

It is also significant that we identified a number of lyso-phospholipids within the CD1d ligand pool. Lyso-phospholipids, particularly LPC, are key signaling molecules generated by enzymatic cleavage of PC by phospholipase A2 molecules, and thus presentation of these molecules by CD1d may serve as an indicator of cellular signaling responses. However, given that the length and structure of the hydrocarbon chains of lipids have been shown to be critical for their stable binding to CD1d molecules [11], and lyso-phospholipids only have a single carbon chain, it is perhaps surprising that that lyso-phospholipids are able to compete effectively with diacylated species for binding to CD1d. Further studies will be required to determine the relative binding affinity of lyso-phospholipids for CD1d, and whether there are specific mechanisms that contribute to the intracellular loading of these lipids.

We also found several glycosylated lipids in the ligand pool, including the terminally sialated species GM3. Since maturation of glycans and terminal sialation occur within the Golgi compartments, this finding suggests either that CD1d molecules can load lipid antigens in the Golgi, or that glycans of CD1d-bound ligands can be modified in the Golgi. Interestingly, two of the glycosylated lipids we identified are tri-hexosylated sphingolipids that bear resemblance to iGb3, a compound identified as a major self antigen for murine NKT cells [21]. We identified the major tri-hexosylated species in the ligand pool as GM3, a compound that does not appear to be highly antigenic for NKT cells [35], but the exact identity of the other species remains undetermined. One of the key antigenic features of iGb3 is that the terminal sugar, a galactose, is linked to the next sugar in an α-anomeric configuration [21]. Humans and other great apes appear during evolution to have lost the glycosyl transferase enzymes required for biosynthesizing this type of glycan on proteins, and it has recently been demonstrated that we also lack a functional iGb3 synthase gene required to synthesize this glycolipid [22]. Hence, despite its possible structural similarity to iGb3, it seems unlikely that the unidentified tri-hexosylated GSL contains the antigenic feature of a terminal galactose residue in an α-linkage.

In conclusion, the results presented here demonstrate that natural cellular ligands of human CD1d molecules include a remarkable variety of lipids. In addition to roles in normal cellular functioning, lipids found in the CD1d ligand pool have been specifically associated with immune recognition in disease. Several, including CL, PE, PI, and gangliosides, are targets of autoantibodies in human autoimmune diseases, and cell surface gangliosides on neoplastic cells can also be targeted by specific antibodies. Thus, an important area for future investigation is to understand how the CD1d-mediated presentation of cellular lipids relates to the immunological targeting of these lipids during disease pathogenesis.

## Materials and Methods

### Generation of secreted human CD1d molecules

To produce secreted CD1d (sCD1d), the cDNA of human CD1d (Genbank accession number NM_001766) was modified by deleting the transmembrane and cytoplasmic domains, and a 30 base-pair tail encoding the 10 amino acid sequence SVVSTDDDLA corresponding to the rat very low density lipoprotein receptor (VLDLr) was added to facilitate purification [36]. sCD1d-VLDLr was cloned into the mammalian expression vector pcDNA3.1 (Invitrogen) and then sequenced to ensure fidelity. The construct was transfected into the HLA class I deficient human B cell line 721.221 as previously described [37]. Transfectants were cultured in an AcuSyst-Maximizer hollow-fiber bioreactor unit (Biovest International, 8500 Evergreen Boulevard, Minneapolis, MN 55433–6000) in RPMI 1640, 10% FBS and 1% penicillin/streptomycin. Cells were monitored for glucose and oxygen consumption. Supernatants containing approximately 25 mg of sCD1d-VLDLr were collected for further analysis.

### Extraction and purification of bound ligands

Clarified supernatant was affinity purified over a Sepharose Fast Flow 4B matrix (Amersham-Pharmacia Biotech, Piscataway, NJ) that was coupled to anti-VLDVr antibody. Bound material was washed with 20 mM sodium phosphate buffer (pH 7.2) and then eluted with 0.2 M acetic acid, pH 2.7, and lyophilized. Ligands were extracted from the CD1d molecules using the chloroform/methanol/water method described by Bligh and Dyer [25]. Lipid classes were separated by normal phase-HPLC (Luna 3 μ Silica (2) 100 Å, 150×2 mm column, Phenomenex, Torrance, CA) using a gradient of n-hexane:2-propanol:water (41:54:5 v/v) and n-hexane:2-propanol:water (37:54:9 v/v), as previously described [38]. Fractions of 200 µL were collected at 1.0 min intervals and monitored by UV absorption at 210 nm.

### Mass spectrometric analysis

The HPLC fractions were sprayed via nanoESI on a Q-STAR quadrupole mass spectrometer with a TOF detector and nanoESI ionization source (PerSceptive SCIEX, Foster City, CA), generating lipid ion maps for each fraction in the range of 350–2000 amu. Ion maps were obtained in both positive and negative ion modes. Ions were manually selected for MS/MS analyses from these ion maps. Lipid structures were determined with the aid of Lipid Maps (www.lipidmaps.org) and published MS studies of phospholipids [39]–[48], sphingomyelins and ceramides [49]–[51], and gangliosides [52]–[55]. Representative synthetic lipids corresponding to each class of lipid were purchased from Avanti Polar Lipids (Alabaster, AL) and subjected to MS/MS under identical collision conditions as the naturally occurring lipid.

### Sialidase treatment

Fraction 33 from the HPLC separation of the CD1d ligand pool was dissolved in aqueous buffer and subject to digestion with sialidase S (Prozyme, San Leandro, CA) according to the manufacturer's protocol. The reaction was then quenched with chloroform, and lipids were extracted from the aqueous phase using chloroform/methanol/water. MS analysis was performed on the organic layer.

## Supporting Information

Figure S1 HPLC analysis of fetal bovine serum. Fetal bovine serum (FBS) or a preparation of affinity purified CD1d were organically extracted according to the method of Bligh and Dyer [25]. The resulting material was separated by normal phase HPLC, resulting in the top trace for the CD1d preparation and the bottom trace for the FBS. The fractions from all peaks that overlapped in the two profiles were analyzed by MS. None of the lipids found in the CD1d fractions were detected in FBS fractions of the corresponding peaks (data not shown), indicating that the CD1d lipids did not derive from the FBS.
(0.24 MB TIF)
