# Supplementary figures and images for: Determination of Cellular Lipids Bound to Human CD1d Molecules
Source: PLoS One. 2009 May 5;4(5):e5325. doi: 10.1371/journal.pone.0005325 (PMC2673035; doi:10.1371/journal.pone.0005325)

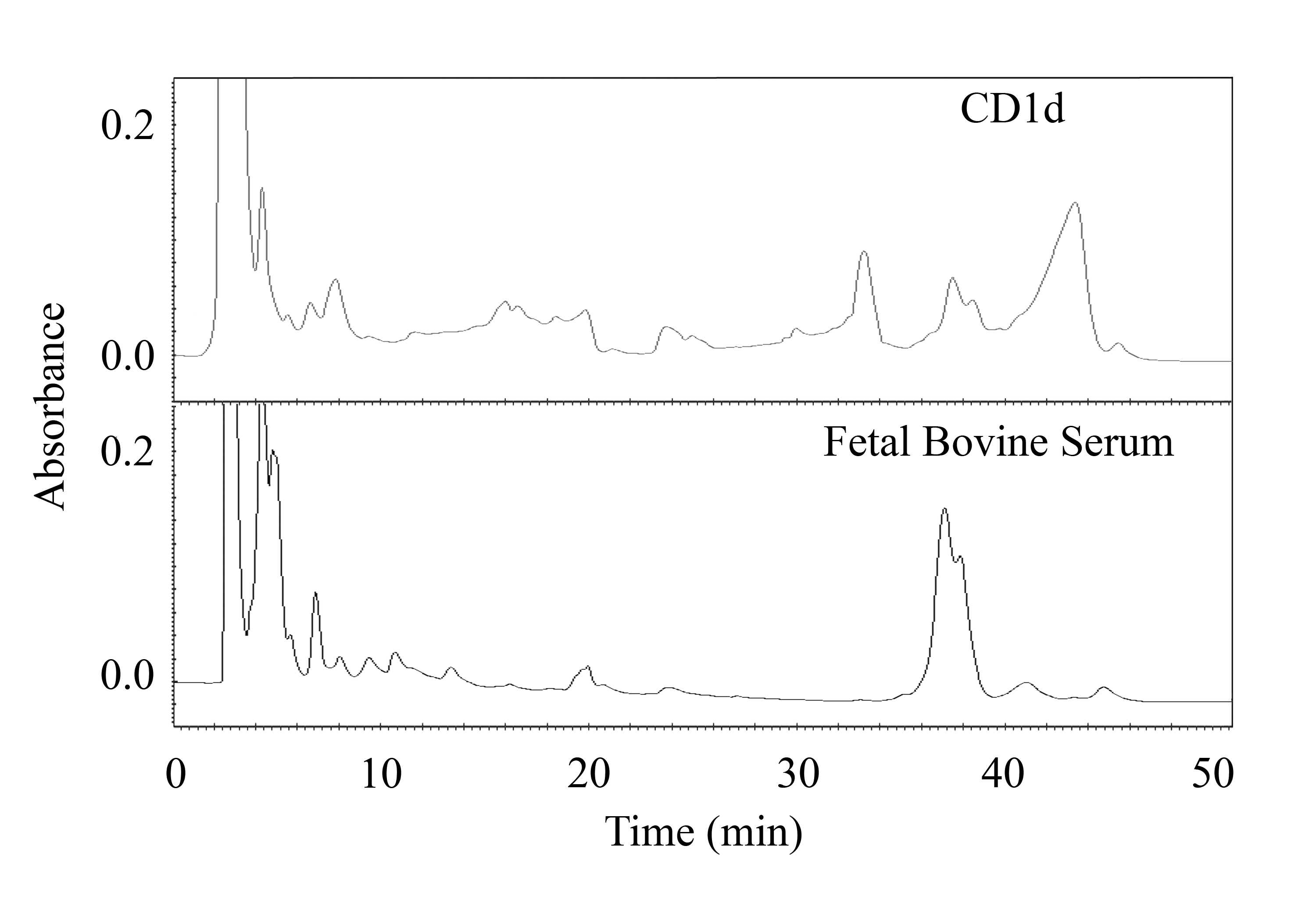

Supplement: Figure S1 — HPLC analysis of fetal bovine serum. Fetal bovine serum (FBS) or a preparation of affinity purified CD1d were organically extracted according to the method of Bligh and Dyer [25]. The resulting material was separated by normal phase HPLC, resulting in the top trace for the CD1d preparation and the bottom trace for the FBS. The fractions from all peaks that overlapped in the two profiles were analyzed by MS. None of the lipids found in the CD1d fractions were detected in FBS fractions of the corresponding peaks (data not shown), indicating that the CD1d lipids did not derive from the FBS. (0.24 MB TIF) [file pone.0005325.s001.tif]
